# The Effects of Physical Exercise on Balance and Prevention of Falls in Older People: A Systematic Review and Meta-Analysis

**DOI:** 10.3390/jcm9082595

**Published:** 2020-08-11

**Authors:** Giuseppe Francesco Papalia, Rocco Papalia, Lorenzo Alirio Diaz Balzani, Guglielmo Torre, Biagio Zampogna, Sebastiano Vasta, Chiara Fossati, Anna Maria Alifano, Vincenzo Denaro

**Affiliations:** 1 Department of Orthopaedic and Trauma Surgery, Campus Bio-Medico University of Rome, 00128 Rome, Italy; r.papalia@unicampus.it (R.P.); l.diaz@unicampus.it (L.A.D.B.); g.torre@unicampus.it (G.T.); b.zampogna@unicampus.it (B.Z.); s.vasta@unicampus.it (S.V.); a.alifano@unicampus.it (A.M.A.); denaro@unicampus.it (V.D.); 2 Department of Movement, Human and Health Sciences, University of Rome “Foro Italico”, 00100 Rome, Italy; chiara.fossati@uniroma4.it

**Keywords:** physical exercise, balance, falls, older people, systematic review, meta-analysis

## Abstract

The aims of this systematic review and meta-analysis were to evaluate the effects of physical exercise on static and dynamic balance in the elderly population, and to analyze the number of falls and fallers. A systematic literature search was conducted using PubMed–Medline, Cochrane Central, and Google Scholar to select randomized clinical trials that analyzed the role of exercise on balance and fall rate in patients aged 65 or older. Sixteen articles were included in this review. Applying the Cochrane risk-of-bias tool, three studies were determined to be at low risk of bias, nine at unclear risk of bias, and four at high risk of bias. The meta-analysis showed improvements in dynamic balance (*p* = 0.008), static balance (*p* = 0.01), participants’ fear of falling (*p* = 0.10), balance confidence (*p* = 0.04), quality of life (*p* = 0.08), and physical performance (*p* = 0.30) in patients who underwent physical exercise compared to controls. The analysis of the total numbers of falls showed a decreased likelihood of falls in patients who participated in exercise programs (*p* = 0.0008). Finally, the number of patients who fell at least once was significantly reduced in the intervention group (*p* = 0.02). Physical exercise is an effective treatment to improve balance and reduce fall rates in the elderly.

## 1. Introduction

Increasingly, attention is being paid by the scientific community to aging, and especially to successful aging. Several interventions aimed to improve the physical and psychosocial status of the elderly have been developed [1]. The decline in physical performance and cognitive capabilities with age causes progressive impairment of muscle strength, coordination, and balance [2], exposing people to a higher risk of falls [3,4]. Human balance is a complex multidimensional concept related to postural control, and it refers essentially to the ability to maintain a posture (e.g., sitting or standing), move between postures, and not fall when reacting to an external disturbance [5]. Apart from the risk of fractures [6] associated with falls, balance represents one of the main features of a plethora of daily activities, both professional and recreational; thus, an impairment of this ability could have a detrimental effect on quality of life [7]. A clear definition has been proposed for a “fall”, namely “inadvertently coming to rest on the ground, floor or other lower level, excluding intentional change in position to rest in furniture, wall or other objects” [8]. Almost one out of three elderly people experience a fall every year [9,10], while a person who experiences at least two falls within 6 months is defined as a “recurrent faller” [11,12]. Falls in older people are concerning events that could result in fractures, residual disability, chronic pain, and loss of independence, leading to important social and public health consequences requiring expensive long-term treatments [13] and accounting for 40% of all injury-related deaths in this group. The severity of the injuries derived from falls vary considerably from minor cutaneous injuries to major fractures, and, in some cases, to fatal traumas [14]. For those patients affected by osteoporosis, the risk of femoral fractures or vertebral body fractures is high, especially for ground-level falls or falls on stairs. The risk for head trauma is consistent across the whole elderly population, and such injuries may result in intracranial pathology with functional sequelae [15]. Progressive physical impairment can happen because of inactivity, which is recognized to be a factor in decreased body function in the elderly [16,17]. Indeed, physical exercise (PE) plays a fundamental role in the prevention of several age-related pathologies, such as metabolic and cardiovascular disease, cancer, and loss of bone quality, to such an extent that the proclamation “exercise is medicine” has been made [18,19]. There is overwhelming evidence that physical exercise can lower the risk of falling in elderly people, averting muscle mass reduction, and improving balance control. In particular, leg strength training seems to be crucial in preventing falls, as lower-limb weakness has been identified as a significant risk factor for falling [20]. In particular, the risk of falling can be assessed using postural control markers. For instance, it has been demonstrated that the risk of a fall is more than doubled when the timed up-and-go (TUG) test requires ≥13.5 s to be completed, the gait speed is <1 m/s, and the modified Romberg test shows a standing time of ≤19 s [21,22]. Furthermore, balance training (BT) has been investigated in recent years [23,24] as an important intervention to slow the physiological decline of balance control in the elderly, and has been revealed to be an effective option for improving balance and postural control [25,26]. Aging involves some changes that affect balance; these include rigidity and reduced range of articular motion, sarcopenia and impaired muscle strength [27,28] cognitive decline, and changes in sensory systems, such as poor vision and hearing [29]. Thus, many systematic reviews and meta-analyses have reported that BT plays a crucial role and it is recommended among other interventions to reduce the risk and rate of falls in older adults [24,30]. We present an updated systematic review and meta-analysis with the aim to analyze the effect of physical exercise on static and dynamic balance in patients aged 65 years or over. The primary endpoint was the improvement of balance performance after various types of physical exercise. The secondary endpoint was the number of falls and/or fallers before and after a course of physical exercise.

## 2. Materials and Methods

This systematic review was conducted in accordance with the Preferred Reporting Items for Systematic Reviews and Meta-Analysis (PRISMA) guidelines [31] and was performed using the PRISMA checklist (Table S1). In this manuscript, we included randomized clinical trials (RCTs) that evaluated the effect of physical exercise on static and dynamic balance and on the number of falls and fallers in the elderly.

### 2.1. Inclusion Criteria

The inclusion criteria were RCTs in the English language published in the past decade, which analyzed the effects of land-based or aquatic exercise on balance and falls rate on patients aged 65 or older, according to the World Health Organization (WHO) definition of the elderly. Exclusion criteria were studies that involved patients with Parkinson’s or Alzheimer’s disease, dementia, hemiplegia, cancer, fibromyalgia, or following a stroke, spinal cord injuries, or fractures. We excluded studies that evaluated activities such as tai chi, yoga, pilates, or dance.

### 2.2. Search Methods

A systematic literature search was conducted using the following online databases: PubMed–Medline, Cochrane Central, and Google Scholar. We used the following search strings: (“Balance” (Journal) OR “balance”(All Fields)) AND (“exercise”(MeSH Terms) OR “exercise”(All Fields) OR (“physical”(All Fields) AND “activity”(All Fields)) OR “physical activity”(All Fields)) AND (“aged”(MeSH Terms) OR “aged”(All Fields) OR “elderly”(All Fields)). Moreover, the following filters were used: randomized controlled trial (article types); 2010/01/01 to 2020/05/01 (publication dates); aged: 65+ years (ages); English (languages); humans (species). The reference lists of the included RCTs were checked in order to select further studies for inclusion. After duplicates were removed, two reviewers (G.P. and S.V.) independently read the abstracts of studies appropriate for inclusion. Differences of opinion were resolved by discussion with the third review author (R.P.). Finally, the full articles were checked by two investigators (G.P. and S.V.) in order to choose the studies to be included in the review and meta-analysis.

### 2.3. Data Collection, Analysis, and Outcomes

Two review authors (G.P. and S.V.) independently performed data extraction. The following data were extracted from the included studies: authors, year of publication, type of study, level of evidence, numbers of participants and their age and sex in both study and control groups, previous falls, follow-up, and intervention(s) in the experimental and in the control group. Many outcomes were analyzed for the assessment of static and dynamic balance, participants’ fear of falling, physical performance, quality of life, and risk of falls. Finally, total number of falls, number of fallers, and fall rate (falls per person-year) were compared between exercise and control groups.

### 2.4. Risk of Bias Assessment

The risk of bias of the included RCTs was independently assessed by two investigators (G.P. and S.V.) by the Cochrane risk-of-bias tool [32]. This tool consists of seven items: random sequence generation, allocation concealment, blinding of participants and personnel, blinding for outcome assessment, incomplete outcome data, selective reporting, and other sources of bias. Each item was graded as having a low, unclear, or high risk of bias. Thus, the trials presented low risk of bias if six or seven domains were reported to have low risk of bias, unclear risk of bias if four of five domains were at low risk of bias, or high risk of bias if fewer than four domains were at low risk of bias.

### 2.5. Statistical Analysis

A meta-analysis was conducted using the Review Manager (RevMan) software Version 5.3. Continuous outcomes were used to assess effects on static and dynamic balance, fear of falling, physical performance, quality of life, and risk of falls between the experimental and the control groups. Dichotomous outcomes were used to assess the total number of falls and the number of fallers between the two groups. Due to the use of different scores for the various outcomes, the continuous data are shown as standard mean difference (SMD) with 95% confidence intervals. Negative values of SMD indicate a benefit for the intervention group. Dichotomous data are presented as odds ratio (OR) with 95% confidence intervals. For the calculation of the weight of the samples of the trials, falls or fallers per month of follow-up were used instead of the total events. The I^2^ test was used to evaluate heterogeneity. In the presence of low heterogeneity (I^2^ < 55%), we used a fixed-effect model; otherwise, a random-effect model was applied. The statistical significance of the results was set at *p* < 0.05

### 2.6. Quality Assessment

The GRADE (Grading of Recommendations Assessment, Development, and Evaluation) assessment was used to evaluate the quality of the evidence of the outcomes and strength of recommendation [33]. This tool evaluates five items for each outcome: risk of bias, inconsistency, indirectness, imprecision, and publication bias. Each component was classified as not serious, serious, or very serious. The GRADE allocates the quality of evidence for the outcomes as high, moderate, low, or very low. RCTs were assigned an initial ranking of high, which could be downgraded for the items mentioned above.

## 3. Results

### 3.1. Results of the Search

**Figure 1:**
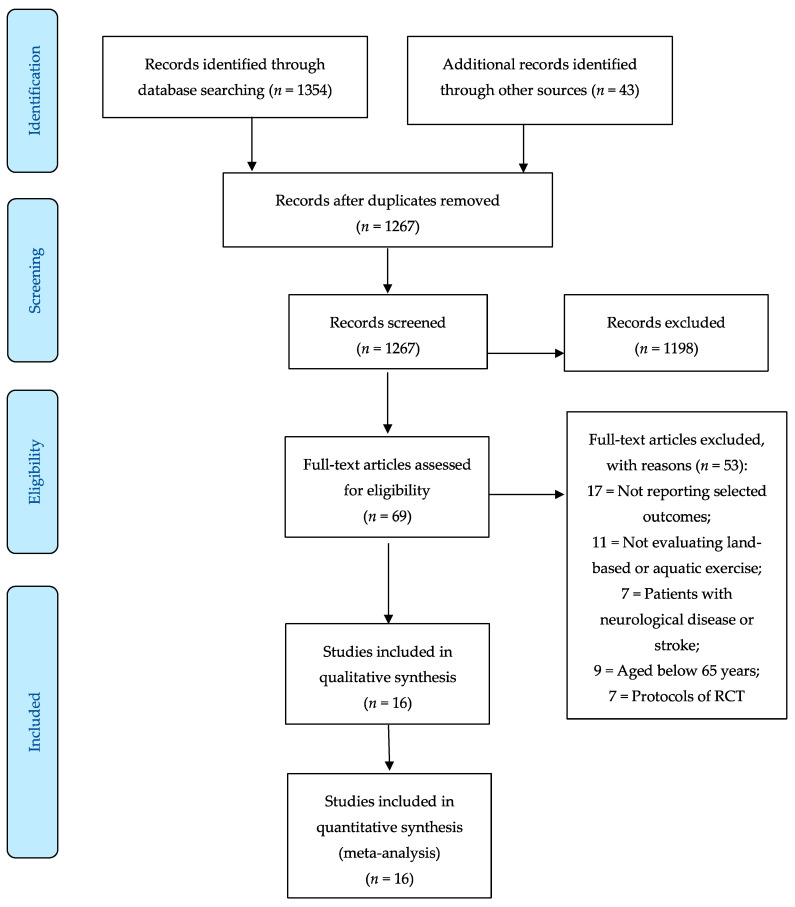
Preferred Reporting Items for Systematic Review and Meta-Analysis (PRISMA) flow diagram.

The literature search yielded 1397 articles. After the removal of duplicates, the titles and abstracts of 1267 of these were examined, leading to the selection of 69 eligible papers that were read in full. Subsequently, 53 studies were excluded for the following reasons: not reporting selected outcomes (*n* = 17), not evaluating land-based or aquatic exercise (*n* = 11), patients aged below 65 years (*n* = 9), protocols of RCT (*n* = 7), and case reports (*n* = 2). Finally, 16 articles were included in this review and meta-analysis (Figure 1).

### 3.2. Demographic Data

The total number of participants in all the included studies was 2960, of which 1521 were in the combined exercise group and 1439 in the combined control group. The ages of the patients ranged from 67.3 to 86 years in the intervention groups, and from 67.2 to 86 in the control groups. The percentages of women in the trials ranged from 50% to 100% in the experimental groups and from 44% to 100% in the control groups. These data indicate a higher inclusion of females for the evaluation of the reported outcomes, and there were four studies that included only elderly women. The percentage of patients that had fallen at least once in the previous year was heterogeneous between the studies, ranging from 13.6% to 62% in the exercise groups and from 12.4% to 69% in the control groups. The demographic characteristics of the patients at baseline are reported in Table 1.

### 3.3. Physical Activity Program

Fourteen studies evaluated land-based exercise, while the remaining two studies [34,35] examined aquatic exercise (Table 2). Regarding exercise protocols, the patients in three trials participated in the Otago Exercise Program (OEP) [36,37,38], while in the other studies, the patients participated in different types of strength and balance training exercise programs. The mean follow-up was 9.2 months and ranged from 4 weeks to 2 years.

### 3.4. Clinical Outcome Data

The mean outcome measures before and after treatment are reported in Table 3. Dynamic balance was assessed in nine studies, using timed up-and-go (TUG) test [35,37,38,39,40,41], TUG-motor [42], TUGcog [34], or 8 foot up-and-go test [43]. Static balance was evaluated via Berg balance score (BBS) in six studies [34,37,40,41,43,44]. Participants’ fear of falling was assessed in five studies, using the Falls Efficacy Scale—International (FES-I) [39,45], Modified Falls Efficacy Scale (MFES) [46], Falls Efficacy Scale, Swedish version (FES(S)) [36], and Thai Falls Efficacy Scale (Thai FES-I) [37]. Balance confidence was reported in four studies, using the Activities-Specific Balance Confidence (ABC) Scale [34,35,46] and short-ABC [47]. Quality of life was assessed in three studies using the Short-Form-36 (SF-36) [39,45] or Short-Form-12 (SF-12) [40] Health Survey. Physical performance was evaluated in three studies, using the Short Physical Performance Battery (SPPB) [36,38,45].

### 3.5. Methodological Evaluation

Upon applying the Cochrane risk-of-bias tool, three studies (18.75%) were determined to be at low risk of bias (A), nine studies (56.25%) were at unclear risk of bias (B), and four studies (25%) were at a high risk of bias (C) (Table 4). More specifically, random sequence generation was adequate in all the studies except one (93.75%). Allocation concealment was considered adequate in 14 trials (87.5%). Blinding for participants and personnel appeared to be impossible due to the nature of the intervention; thus, it was inadequate in 15 studies (93.75%). Blinding for outcome assessment was graded as adequate in 13 studies (81.25%). Incomplete outcome data were judged as adequate in eight studies (50%). Selective reporting was considered adequate in 11 studies (68.75%). Other sources of bias were adequate in eight trials (50%).

### 3.6. Effect of Intervention

**Figure 2:**
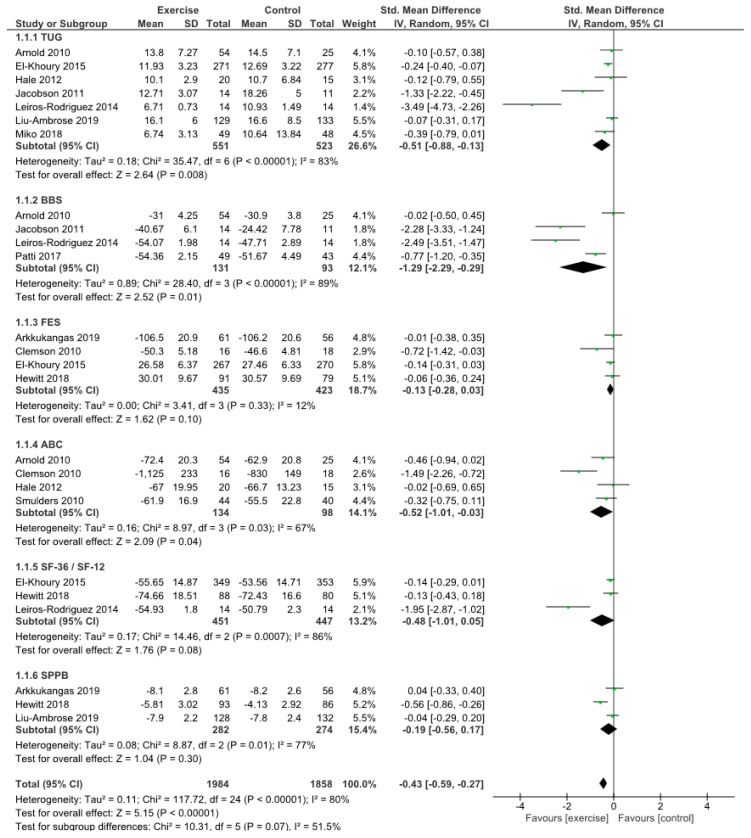
Outcome measurements.

The meta-analysis showed the effect of exercise on dynamic balance, static balance, participants’ fear of falling, balance confidence, quality of life, and physical performance compared to controls (Figure 2). TUG times decreased significantly in the intervention group, demonstrating significant improvements in dynamic balance in comparison with the control group (SMD −0.51, 95% CI −0.88 to −0.13, *p* = 0.008). BBS showed significant improvements in static balance for the exercise group (SMD −1.29, 95% CI −2.29 to −0.29, *p* = 0.01). FES showed better fear-of-falling outcomes in patients who did physical exercise compared to controls (SMD −0.13, 95% CI −0.28 to 0.03), but no significant differences (*p* = 0.10). Balance confidence, assessed by ABC, showed significant differences in favor of the experimental group (SMD −0.52, 95% CI −1.01 to −0.03, *p* = 0.04). Short-Form Health Survey results showed greater improvements in quality of life in the experimental groups (SMD −0.48, 95% CI −1.01 to −0.05), without significant differences (*p* = 0.08). SPPB showed no significant differences in physical performance between the two groups (*p* = 0.30), but better outcomes in the exercise group (SMD −0.19, 95% CI −0.56 to 0.17). Summarily, analyzing all the reported scores, a significant difference was shown in favor of the physical exercise group compared to the controls (*p* > 0.00001).

**Figure 3:**
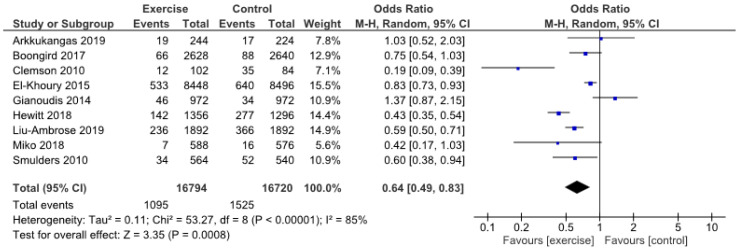
Total number of falls.

The analysis of the total numbers of falls showed a statistically significant decreased likelihood of falls in patients who participated in exercise programs (OR 0.64, 95% CI 0.49 to 0.83, *p* = 0.0008) (Figure 3).

**Figure 4:**
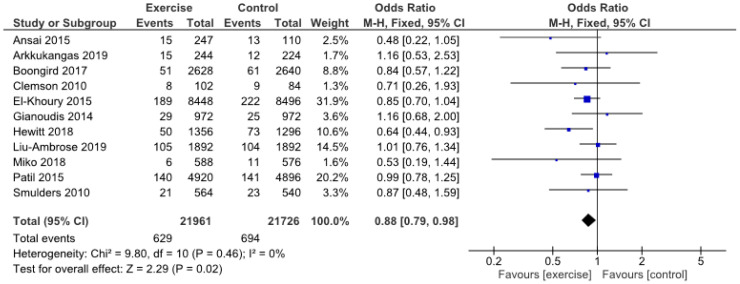
Number of fallers (≥1 falls).

Finally, the number of patients who fell at least once was significantly reduced in the intervention group compared to the control group (OR 0.88, 95% CI 0.79 to 0.98, *p* = 0.02) (Figure 4).

### 3.7. Quality Assessment

The GRADE was used to assess the quality of the evidence provided in the included trials (Table 5). There were six comparisons for continuous data and two for dichotomous data. Regarding scores, TUG and BBS were downgraded by one level due to inconsistency of the results; thus, they reported a moderate quality. FES, ABC, and SPPB showed moderate quality because they were downgraded by one level for serious risk of bias. Finally, SF-36/SF-12 was downgraded by two levels due to serious risk of bias and inconsistency; thus, it presented low quality. In contrast, the outcomes of both total number of falls and fallers maintained a high quality of evidence.

## 4. Discussion

The primary aim of this systematic review and meta-analysis was to evaluate the improvement of balance performance in the elderly population after various types of physical exercise. A secondary endpoint analyzed was the number of falls and/or fallers before and after an exercise program. Physical exercise was shown to be very beneficial for older people in terms of dynamic and static balance, fear of falling, balance confidence, quality of life, and physical performance, with a significant improvement reported for all the considered scores in patients who participated in a physical treatment compared to controls. The meta-analysis proved that the parameters for dynamic and static balance, such as TUG and BBS, demonstrated the best improvements after PE, with the most statistical significance compared to controls. In fact, balance training led to higher confidence in the participants’ ability to perform various daily activities without falling, better patient mobility and safety at speed, greater ability to perform balance-related tasks, and lessened difficulties with activities of daily living. PE seemed to be particularly useful in reducing falls via the increase of postural control, more than improving quality of life and physical performance in older people. Moreover, with the exception of SF-36/SF-12 which was low, all the other outcomes presented a moderate quality of evidence as assessed by GRADE, thus supporting a recommendation of physical exercise in the geriatric population with risk of falls. The number of falls was recorded using daily fall calendars, which were returned to the blinded investigators. Regarding the evaluation of the number of falls and fallers, almost all the studies showed great improvements in the patients who underwent PE. Furthermore, the data showed both high quality and strength of recommendation according to GRADE for the benefit of balance and postural control exercises to reduce the rate of falls in the elderly. Only one study [48] reported no benefit in terms of falls and fallers after the exercise program, although it represented an effective approach for the improvement of multiple musculoskeletal and functional performance in older adults with risk factors for falls. Moreover, the meta-analysis showed that in patients who performed physical exercise, there was a statistically significant decrease in both the number of falls and fallers (respectively *p* = 0.0008 and *p* = 0,02). Furthermore, PE was more effective in reducing the total number of falls than the number of fallers, showing that improving muscle tropism and postural balance through specific protocol of exercises reduced the risk of falls, but it did not completely eradicate the risk of falling at least once. These data seem to strengthen the concept that PE represents a crucial aspect of prevention for reducing the risk of falls, which can lead to fractures and consequently to hospitalization, surgical procedures, and prolonged immobilization, with an increase in national healthcare costs. In contrast, a study by Lee et al. analyzed the effectiveness of exercise interventions on the rate of falls and number of fallers in care facilities, and they showed significant differences between all exercise interventions and control groups in the rate of falls, but they did not find differences in the number of fallers between all exercise interventions and control groups [50]. Similarly, Zhao et al. [51] showed that exercise had a positive effect on the reduction of fall-related fractures, with improvements in the rate of falls and leg strength in older people; however, they reported only a marginally beneficial effect of exercise on balance. Although the study population was represented by elderly people, the mean ages of the patients were different in the various studies. However, there was not a marked correlation found between age and greater improvements in the outcomes after balance training. Moreover, four studies enrolled only women, while the others included both sexes at various percentages. It seemed that in studies with only women, there were better improvements in the selected outcomes; therefore, the role of physical exercise and balance training could be greater in elderly women, and in the prevention of osteoporosis, reducing the risk of fracture following falls. In this systematic review, we included only RCTs in order to evaluate the role of physical activity compared to usual care. However, it was not possible to compare different kinds of physical activity in order to determine which is better for older people. In particular, only two studies analyzed aquatic therapy [34,35], which seems to be an interesting alternative to land-based exercise for the geriatric population, permitting low-impact and low-weight-bearing exercise. In fact, Guillamon et al. [52] presented some evidence that aquatic exercise can improve modifiable risk factors of falls, although the quality of this evidence was low, and there was a lack of consistency between studies. A limitation of this systematic review was the high variability of the training protocols of the included studies. Only few studies reported a fall-prevention program with a standardized protocol. The Otago Exercise Program was applied in three studies [36,37,38]; this is an individualized home-based program of balance and strength retraining exercises designed specifically to prevent falls [53]. Hewitt et al. [45] tested the Sunbeam program, which consists of individually prescribed progressive resistance training plus balance exercise. The Ossébo intervention, used by El-Khoury et al. [39], is composed of exercises designed to improve postural stability, muscle extensibility, balance, reaction time, coordination, and internal sense of spatial orientation [54]. The Lifestyle Approach to Reducing Falls through Exercise (LiFE) program, reported on by Clemson et al. [46], is a home-based, lifestyle-integrated balance and muscle-strengthening exercise training program specifically developed for fall prevention. Otherwise, the other studies did not refer to standardized exercise protocols, but they reported various generic exercises for postural balance, stationary strengthening [43], weight bearing [49], aerobic elements [41], and proprioceptive training and eye–hand/eye–foot coordinative [44]. Finally, Leiros-Rodríguez et al. [40] evaluated balance exercises as a recreational activity in public parks. Therefore, quite all the studies differed in terms of the applied exercise program; thus, it was difficult to compare the different results for the balance scores. Moreover, there was high heterogeneity among the studies in terms of the volume of training, due to different numbers of training weeks, sessions per week, exercises per session, repetitions per set, and sets per exercise, which resulted in varying loads of balance and strength training and fall-prevention exercises. Another limitation was the relatively short follow-up of some studies: it was one year or less in 87.5% of the studies, and may not allow the long-term effects of PA on dynamic and static balance to be determined. However, the overall quality of the included studies was good, with only four studies at a high risk of bias. The only critical item regarded the blinding for participants and personnel, which was at a high risk of bias in all the studies except one [49], but this was comprehensible for RCTs that compared clinical outcomes and the details of falls in patients who participated in specific exercise programs or no intervention. Instead, almost all studies used a clearly randomized allocation sequence and concealment, and the outcomes and falls were classified by qualified examiners (e.g., geriatricians or physical therapists) blinded to group assignment. Furthermore, the adherence to treatment in the studies was high and the patients lost to follow-up were few, demonstrating that PE protocols were well accepted by patients, despite their elderly age. In the literature search, we excluded many studies because they evaluated the role of PE only in patients with neurological or cardiovascular disease, whereas we were interested in analyzing the importance of exercise in the entire geriatric population, especially in healthy people, in order to limit the reduction of postural control and loss of muscle strength that predispose this population to an increased risk for falls [1]. We also focused on the differences between studies regarding the percentage of patients that had fallen at least once in the previous year. These data varied between 12.4% and 69% in the pretreatment measurements. Moreover, in many studies, this percentage was higher in the study group than in the control group; therefore, the result of fewer falls and fallers at follow-up in the study group acquires even more significance in light of these considerations. Valdés-Badilla et al. [55] reported the beneficial effects on quality of life, fall risk, activities of daily living, physical activity levels, and blood parameters of governmental physical activity programs for independent older adults. Finally, Tricco et al. [56] demonstrated that exercise was associated with a lower risk of injurious falls compared with usual care, but the type of physical activity used to reduce falls should be selected on the basis of patient and caregiver values. Sherrington et al. [24], in their systematic review and meta-analysis, demonstrated that a high dose of exercise, particularly involving balance training, can prevent falls in older people. In a more recent meta-analysis [57], the same group showed that exercise reduced the rate of falls in community-dwelling older people by 21%, and had a fall-prevention effect in community-dwelling people with Parkinson’s disease or cognitive impairment. In contrast to these studies, we evaluated as outcomes not only the rate of falls or number of fallers, but also the clinical scores for dynamic and static balance, participants’ fear of falling, physical performance, and quality of life in order to analyze the improvements in performing daily activities with less risk and fear of falling. Moreover, we focused on elderly people without neurological or cardiovascular disease, to recommend PE as an effective treatment for all the elderly population which could prevent impairment of muscle strength and a higher likelihood of falling. Furthermore, we analyzed various kinds of specific BT programs, including aquatic exercise, which could represent valid alternative or complementary activity to land-based exercise.

## 5. Conclusions

This systematic review proved that physical exercise is an effective treatment to improve static and dynamic balance and reduce the number of falls and fallers for patients aged 65 or over. The meta-analysis reports strong evidence that exercise programs can reduce fall rates in the geriatric population. Balance and postural exercises should be included in training protocols for the elderly in order to prevent the risk of falls, and they should be performed in the entire healthy population, not only as rehabilitation after stroke, fractures, or for patients affected by neurodegenerative disease. However, further large-scale trials with longer follow-up are needed to estimate the long-term effects of balance programs on decreasing the rate of falls. Moreover, more studies involving aquatic exercise or comparing aquatic versus land-based programs are necessary to promote innovative strategies to prevent falls in older people, which can be delivered by exercise trainers.

## Figures and Tables

**Table 1:** Demographic characteristics of the patients at baseline.

Author (Year)	Type of Study	LOE	Study Group			Control Group			Fell at least Once in Past Year	
			*n*	Age	Sex	*n*	Age	Sex	Study	Control
Ansai et al., (2015) [42]	RCT	I	23	81.9 y	73.9% F, 26.1% M	23	82.6 y	65.2% F, 34.8% M	10 (43.5%) *	8 (34.8%) *
			23	82.8 y	65.2% F, 34.8% M				7 (30.4%) *	
Arkkukangas et al., (2019) [36]	RCT	I	61	83 y	67% F, 33% M	56	82 y	73% F, 27% M	24 (39%)	21 (37%)
Arnold et al., (2010) [34]	RCT	I	28	73.2 y	71.4% F, 28.6% M	25	75.8 y	64%F, 36%M	14 (50%)	9 (36%)
			26	74.4 y	77% F, 23% M				16 (62%)	
Boongird et al., (2017), [37]	RCT	I	219	74.08 y	83.6% F, 16.4% M	220	73.94 y	81.4% F, 18.6% M		
Clemson et al., (2010) [46]	RCT	I	18	81 y	50% F, 50% M	16	82 y	44% F, 56% M	7 (39%) †	4 (25%) †
El-Khoury et al., (2015) [39]	RCT	I	352	79.8 y	100% F	354	79.6 y	100% F	137 (39%)	159 (45%)
Gianoudis et al., (2014) [48]	RCT	I	81	67.7 y	64% F, 36% M	81	67.2 y	73% F, 27% M	11 (13.6%)	10 (12.4%)
Hale et al., (2012) [35]	RCT	I	23	73.6 y	74% F, 26% M	16	75.7 y	75% F, 25% M	13 (61%)	11 (69%)
Hewitt et al., (2018) [45]	RCT	I	113	86 y	62.8% F, 37.2% M	108	86 y	68.2% F, 31.8% M	69 (61%)	54 (50.5%)
Jacobson et al., (2011) [43]	RCT	I	14	83.05 y	57.1% F, 42.9% M	11	81.37 y	72.7% F, 27.3% M		
Leiros-Rodriíguez et al., (2014) [40]	RCT	I	14	69 y	100% F	14	68 y	100% F		
Liu-Ambrose et al., (2019) [38]	RCT	I	172	81.2 y	64% F, 36% M	172	81.9 y	69% F, 31% M	43 (25%)	60 (35%)
Miko et al., (2018) [41]	RCT	I	50	69.33 y	100% F	50	69.10 y	100% F		
Patil et al., (2015) [49]	RCT	I	205	74.4 y	100% F	204	74 y	100% F		
Patti et al., (2017) [44]	RCT	I	49	67.32 y	53% F, 47% M	43	68.93 y	55.8% F, 44.2% M		
Smulders et al., (2010) [47]	RCT	I	50	70.5 y	90% F, 10% M	46	71.6 y	97.8% F, 2.2% M		

RCT: randomized clinical trial; LOE: levels of evidence; N.: number of participants; y: years; F: female; M: male. * = fall in the past 3 months; † = frequent falls (≥3) in the past 12 months.

**Table 2:** Clinical results of the included studies.

Author	Intervention(s)	Control	Follow-up	Results
Ansai	Multicomponent training: protocol consisting of warm-up, aerobic, strength, balance, and cool-down exercises for 16 weeks	No intervention	22 w	There were no significant differences between groups and assessments in any variable.
	Resistance training: leg press, chest press, calf, back extension, abdominal, and rowing for 16 weeks			
Arkkukangas	OEP: home-based exercise program designed to improve strength, balance, and endurance over 12 weeks	No intervention	12 w	In the short-term perspective, there were no benefits of an exercise program regarding physical performance, fall self-efficacy, activity level, hand-grip strength, and fall frequency in comparison to a CG.
Arnold	AE protocol consisted of lower- and upper-extremity strengthening, trunk-control, and balance exercises twice a week for 11 weeks (preceded by educational session in the aquatics and education group)	No intervention	11 w	Significant improvement in fall risk factors (*p* = 0.038) with the combination of aquatic exercise and education.
Boongird	Modified OEP: five combined leg-muscle strengthening, balance retraining, and stretching exercises, which progressed in difficulty, and a walking plan	Fall prevention education	12 m	The incidence of falls was 0.30 falls per person year in the EG, compared with 0.40 in the CG. The fear of falling was significantly lower in the EG than CG (*p* = 0.003).
Clemson	Lifestyle Approach to Reducing Falls through Exercise (LiFE) program: home-based balance and strengthening exercise program for fall prevention	No intervention	6 m	The relative risk analysis demonstrated a significant reduction in falls in EG (RR = 0.23). Dynamic balance improved for the LiFE program participants (*p* = 0.04).
El-Khoury	Ossébo program: weekly supervised group sessions of progressive balance training for two years, supplemented by individually prescribed home exercises	No intervention	24 m	The injurious fall rate was 19% lower in the EG than in the CG (*p* = 0.04). The EG fared significantly better than the CG in all balance and gait performance tests.
Gianoudis	Multimodal program incorporating high-velocity progressive resistance training, weight-bearing impact and/or balance training, and fall-prevention exercises, performed three days per week for 12 months	Standard care self-management	12 m	There were no significant differences in fall incidence between the groups, or in the number of participants sustaining one or more falls or multiple falls.
Hale	Water-based exercise classes twice weekly for 12 weeks	Time-matched computer training program for 12 weeks	12 w	Water-based exercise did not reduce falls risk compared with attending a computer-skills training class.
Hewiit	Resistance training plus balance exercises performed in a group setting for 50 h over a 25-week period (Sunbeam program), followed by a maintenance period for 6 months	Regular activity schedule	12 m	Overall incidence of falls in the EG of 1.31 per person-year, compared with 2.91 in the CG. A significantly greater improvement was found in physical performance in the EG than in the CG (*p* = 0.02).
Jacobson	Standing, static balancing, and mild leg exercise, 12 min per session, three times per week for 12 weeks.	Regular group exercise	12 w	Significant (*p* < 0.01) improvement for the EG over the CG in the 30 s chair test repetitions, 8-foot up and go test, balance assessment, and leg-function assessments.
Leiros-Rodriíguez	12 sessions of balance exercises for 50 min	No intervention	4 w	Berg Balance Scale, timed up-and-go test, and SF-12 showed statistically significant differences in the EG (*p* < 0.05).
Liu-Ambrose	Usual care plus OEP (a home-based strength and balance retraining exercise program) for 12 months	Fall-prevention care	11 m	Fall rates were lower in the EG compared with CG (IRR, 0.64; *p* = 0.009). The estimated fall rate incidence was 1.4 per person-year in the EG and 2.1 in the CG (*p* = 0.006).
Miko	12-month of balance-training exercise program (three times a week for 30 min)	No intervention	12 m	TUG and BBS test scores showed a statistically significant difference between EG and CG (*p* < 0.005). The event rate for the number of patients who fell was 0.122 in the EG and 0.229 in the CG, thus the relative risk of falls was 0.534 (*p* = 0.17).
Patil	Group exercise classes twice a week for 12 months and once a week for the subsequent 12 months and home exercises	Current physical activity	24 m	Timed up-and-go and grip strength did not differ between groups. There was no difference in the total falls incidence rate ratio (IRR = 1.0).
Patti	Joint mobility, cardiovascular exercise, strengthening of core stability, proprioceptive training, and eye–hand/eye–foot coordinative exercises for 13 weeks	No intervention	14 w	Only the EG group demonstrated significant improvements in balance skills (*p* < 0.0001).
Smulders	11 sessions over 5.5 weeks of education, obstacle course, walking exercises, weight-bearing exercises, correction of gait abnormalities, and training in fall technique	No intervention	12 m	The fall rate in the exercise group was 39% lower than for the control group (0.72 vs 1.18 falls/person-year; risk ratio of 0.61).

OEP: Otago Exercise Program; AE: aquatic exercise; w: weeks; m: months; CG: control group; EG: experimental group.

**Table 3:** Outcome measures before and after treatment.

Study		TUG (s)		BBS (pts)		FES (pts)		ABC (pts)		SF-36/SF-12 (pts)		SPPB ^9^ (pts)	
		Pre	Post	Pre	Post	Pre	Post	Pre	Post	Pre	Post	Post	Pre
Ansai	I	30.4 ± 12.2	29.8 ± 13.1										
		29.0 ± 15.5	26.7 ± 22.2										
	C	25.3 ± 8.4	25.3 ± 9.3										
Arkkukangas	I					103.3 ± 21.3 ^1^	106.5 ± 20.9					7.9 ± 2.4	8.1 ± 2.8
	C					100.2 ± 26.5	106.2 ± 20.6					7.6 ± 2.5	8.2 ± 2.6
Arnold	I	14.9 ± 5.6	12.6 ± 3.9	30.4 ± 3.8	31.4 ± 3.2			69.2 + 19.9 ^5^	75.0 ± 15.2				
		15.8 ± 9.1	15.1 ± 9.5	29.3 ± 5.2	30.5 + 5.1			70.4 ± 21.9	69.6 ± 24.4				
	C	14.3 ± 6.7	14.5 ± 7.1	31.1 ± 2.7	30.9 + 3.8			65.3 ± 18.1	62.9 ± 20.8				
Boongird	I	16.75	16.7	21.68	21.6	27.11 ^2^	24.7						
	C	16.6	16.8	21.48	21.7	26.84	27						
Clemson	I					45.3 ± 8.2 ^3^	50.3 ± 5.18	925 ± 341 ^6^	1125 ± 233				
	C					45.1 ± 8.7	46.6 ± 4.81	825 ± 372	830 ± 149				
El-Khoury	I	12.38 ± 2.76	11.93 ± 3.23			25.52 ± 7.06 ^4^	26.58 ± 6.37			57.05 ± 15.80 ^7^	55.65 ± 14.87		
	C	12.39 ± 3.09	12.69 ± 3.22			26.02 ± 6.97	27.46 ± 6.33			54.72 ± 16.05	53.56 ± 14.71		
Hale	I	11.0 ± 3.13	10.1 ± 2.9					64.2 ± 19.3 ^5^	67.0 ± 19.95				
	C	10.7 ± 5.78	10.7 ± 6.84					66.4 ± 19.8	66.7 ± 13.23				
Hewitt	I					27.75 ± 10.08 ^4^	30.01 ± 9.67			65.72 ± 18.30 ^7^	74.66 ± 18.51	5.16 ± 2.57	5.81 ± 3.02
	C					31.28 ± 13.03	30.57 ± 9.69			64.96 ± 16.98	72.43 ± 16.60	4.30 ± 2.90	4.13 ± 2.92
Jacobson	I	16.97 ± 5.63	12.71 ± 3.07	26.91 ± 3.65	40.67 ± 6.10								
	C	16.20 ± 3.67	18.26 ± 5	27.24 ± 4.64	24.42 ± 7.78								
Leiros-Rodriíguez	I	11 ± 1.3	6.71 ± 0.73	45.86 ± 2.91	54.07 ± 1.98					49.36 ± 3.2 ^8^	54.93 ± 1.8		
	C	11.14 ± 1.68	10.93 ± 1.49	47.79 ± 3.38	47.71 ± 2.89					50.29 ± 2.5	50.79 ± 2.3		
Liu-Ambrose	I	16.3 ± 7	16.1 ± 6									7.9 ± 2.2	7.9 ± 2.2
	C	16.9 ± 6.4	16.6 ± 8.5									7.8 ± 2.3	7.8 ± 2.4
Miko	I	8.89 ± 7.38	6.74 ± 3.13										
	C	9.95 ± 12.02	10.64 ± 13.84										
Patti	I			51.83 ± 4.17	54.36 ± 2.15								
	C			51.09 ± 3.89	51.67 ± 4.49								
Smulders	I							54.3 ± 19.7 _5_	61.9 ± 16.9				
	C							55.5 ± 22.1	55.5 ± 22.8				

I: intervention group; C: control group; TUG: timed up-and-go; BBS: Berg Balance Score; FES: Falls Efficacy Scale; ABC: Activities-Specific Balance Confidence; SF-36/SF-12: Short Form-36/Short Form-12 Health Survey; SPPB: Short Physical Performance Battery; s: seconds; pts: points. FES: ^1^ = FES Swedish version (0–130); ^2^ = Thai Falls Efficacy Scale (16–64); ^3^ = Modified Falls Efficacy Scale (0–140); ^4^ = FES-I (16–64); ABC: ^5^ = ABC (0–100); ^6^ = ABC (0–1600); SF-36: ^7^ = SF-36 (0–100); SF-12: ^8^ = SF-12 (0–100); SPPB: ^9^ = SPPB (0–12).

**Table 4:** Cochrane risk-of-bias tool for randomized controlled trials.

Study	Random Sequence Generation	Allocation Concealment	Blinding (Participants and Personnel)	Blinding (Outcome Assessment)	Incomplete Outcome Data	Selective Reporting	Other Sources of Bias	Risk of Bias
Ansai	L	L	H	L	L	H	U	B
Arkkukangas	L	L	H	L	L	L	U	B
Arnold	L	L	H	L	L	U	L	B
Boongird	L	L	H	L	U	L	L	B
Clemson	L	L	H	L	H	L	U	B
El-Khoury	L	L	H	L	L	L	L	A
Gianoudis	L	U	H	U	U	L	L	C
Hale	L	L	H	L	U	L	L	B
Hewitt	L	L	H	L	L	L	L	A
Jacobson	L	L	H	H	L	H	L	B
Leiros-Rodriíguez	L	U	H	U	L	L	U	C
Liu-Ambrose	U	L	H	L	L	L	L	A
Miko	L	L	H	L	H	L	U	B
Patil	L	L	U	L	H	U	U	C
Patti	L	L	H	L	H	U	U	C

L: low; U: unclear; H: high.

**Table 5:** GRADE

Outcomes	Number of Participants (Studies)	Risk of Bias	Inconsistency	Indirectness	Imprecision	Other Considerations	Quality
TUG	1074 (7 RCTs)	not serious	serious	not serious	not serious	not serious	⨁⨁⨁◯ moderate
BBS	224 (4 RCTs)	not serious	serious	not serious	not serious	not serious	⨁⨁⨁◯ moderate
FES	858 (4 RCTs)	serious	not serious	not serious	not serious	not serious	⨁⨁⨁◯ moderate
ABC	232 (4 RCTs)	serious	not serious	not serious	not serious	not serious	⨁⨁⨁◯ moderate
SF-36/SF-12	898 (3 RCTs)	serious	serious	not serious	not serious	not serious	⨁⨁◯◯ low
SPPB	556 (3 RCTs)	serious	not serious	not serious	not serious	not serious	⨁⨁⨁◯ moderate
Total number of falls	2158 (9 RCTs)	not serious	not serious	not serious	not serious	not serious	⨁⨁⨁⨁ high
Number of fallers (≥1 falls)	2632 (11 RCTs)	not serious	not serious	not serious	not serious	not serious	⨁⨁⨁⨁ high

TUG: timed up-and-go; BBS: Berg Balance Score; FES: Falls Efficacy Scale; ABC: Activities-Specific Balance Confidence; SF-36/SF-12: Short Form-36/Short Form-12 Health Survey; SPPB: Short Physical Performance Battery; RCT: randomized clinical trial.

## Supplementary Materials

The following are available online at https://www.mdpi.com/2077-0383/9/8/2595/s1, Table S1: PRISMA Checklist.
